# Caspase-6 promotes activation of the caspase-11-NLRP3 inflammasome during gram-negative bacterial infections

**DOI:** 10.1016/j.jbc.2021.101379

**Published:** 2021-11-02

**Authors:** Min Zheng, Rajendra Karki, Balabhaskararao Kancharana, Hartmut Berns, Shondra M. Pruett-Miller, Thirumala-Devi Kanneganti

**Affiliations:** 1 Department of Immunology, St Jude Children's Research Hospital, Memphis, Tennessee, USA; 2 Center for Advanced Genome Engineering, St Jude Children's Research Hospital, Memphis, Tennessee, USA

**Keywords:** caspase-6, caspase-11, inflammasome, NLRP3, gasdermin D, pyroptosis, cell death, gram-negative bacteria, *E. coli*, *C. rodentium*, *P. aeruginosa*, ASC, apoptosis-associated speck-like protein containing a CARD, BMDMs, bone marrow-derived macrophages, CARD, caspase-activation and recruitment domain, CASP11, caspase-11, CASP6, caspase-6, ERK, extracellular signal-regulated kinase, GBP, guanylate-binding proteins, GSDMD, gasdermin D, IAV, influenza A virus, IFN, interferon, IRGB10, immunity-related GTPase B10, LPS, lipopolysaccharide, NGS, next generation sequencing, NLR, nucleotide-binding oligomerization domain-like receptor, NLRP, nucleotide-binding oligomerization domain-like receptor containing pyrin domain

## Abstract

The innate immune system acts as the first line of defense against infection. One key component of the innate immune response to gram-negative bacterial infections is inflammasome activation. The caspase-11 (CASP11)-nucleotide-binding oligomerization domain-like receptor pyrin domain-containing 3 (NLRP3) inflammasome is activated by cytosolic lipopolysaccharide, a gram-negative bacterial cell wall component, to trigger pyroptosis and host defense during infection. Although several cellular signaling pathways have been shown to regulate CASP11-NLRP3 inflammasome activation in response to lipopolysaccharide, the upstream molecules regulating CASP11 activation during infection with live pathogens remain unclear. Here, we report that the understudied caspase-6 (CASP6) contributes to the activation of the CASP11-NLRP3 inflammasome in response to infections with gram-negative bacteria. Using *in vitro* cellular systems with bone marrow-derived macrophages and 293T cells, we found that CASP6 can directly process CASP11 by cleaving at Asp59 and Asp285, the CASP11 auto-cleavage sites, which could contribute to the activation of CASP11 during gram-negative bacterial infection. Thus, the loss of CASP6 led to impaired CASP11-NLRP3 inflammasome activation in response to gram-negative bacteria. These results demonstrate that CASP6 potentiates activation of the CASP11-NLRP3 inflammasome to produce inflammatory cytokines during gram-negative bacterial infections.

The inflammasome is a multiprotein complex which canonically consists of a sensor, the adapter molecule apoptosis-associated speck-like protein containing a caspase-activation and recruitment domain (CARD) (ASC) and the inflammatory caspase, caspase-1 (1, 2). To date, five well-studied inflammasomes have been established, including the nucleotide-binding oligomerization domain-like receptor (NLR) family, pyrin domain-containing 1 (NLRP1), NLRP3, NLR-family CARD-containing 4 , absent in melanoma 2, and pyrin inflammasomes. Among these, the NLRP3 inflammasome is the best characterized and can be activated by a variety of stimuli (1, 3). NLRP3 inflammasome activation has been classified as canonical and noncanonical depending on the involvement of caspase-11 (CASP11; or its human counterpart caspase-4/5). The noncanonical NLRP3 inflammasome is assembled upon CASP11 sensing of cytosolic lipopolysaccharide (LPS). CASP11 then undergoes oligomerization mediated by its CARD domain and proximity-induced activation (4, 5, 6, 7). After activation, CASP11 directly processes gasdermin D (GSDMD) to release its active N-terminus, which forms pores in the plasma membrane (8, 9). These GSDMD-mediated pores allow for changes in ion flux in the cell, which leads to the assembly of the NLRP3 inflammasome (8). Here we refer to this noncanonical NLRP3 inflammasome activation as the formation of the CASP11-NLRP3 inflammasome.

Several host factors have been identified in the regulation of CASP11-NLRP3 inflammasome activation. TIR-domain-containing adapter-inducing interferon-β-dependent signaling downstream of toll-like receptor 4 is essential for upregulating the expression of CASP11 (10, 11), although the requirement for TIR-domain-containing adapter-inducing interferon-β can be bypassed upon stimulation with ligands to activate other toll-like receptors, such as Pam3CSK4 (6). In addition, guanylate-binding proteins (GBPs) and immunity-related GTPase B10 (IRGB10) are also involved in gram-negative bacteria–mediated, but not LPS transfection-induced, CASP11-NLRP3 inflammasome activation; these molecules target the bacterial cell wall to release LPS to be sensed by CASP11 (12, 13). GBPs can also target the bacterial-derived outer membrane vesicles to liberate LPS into the cytosol, although the underlying mechanism is not fully understood (13, 14). Another host factor, high mobility group box 1, has also been reported to bind LPS and facilitate uptake by lysosomes and subsequently allow the LPS to be leaked into the cytosol to activate CASP11 (15). Transcription factors can also play key roles in the regulation of the CASP11-NLRP3 inflammasome. The transcription factor IRF8 contributes to the induction of the type I interferon (IFN) response during gram-negative bacterial infection and promotes CASP11-NLRP3 inflammasome activation without affecting the CASP11 expression (16). In human cells, it has been recently reported that the transcription factor IRF2 is required for the expression of CASP4, and deletion of IRF2 dampens CASP4-NLRP3 inflammasome activation (17). Overall, there are fundamental differences in the regulation of CASP11-NLRP3 inflammasome activation in response to gram-negative bacterial infection compared with its regulation during LPS transfection.

In addition to these factors that regulate the expression of CASP11 or mediate the release of LPS for sensing, other host proteins are involved in regulating the NLRP3 inflammasome more broadly. The key examples include FADD and CASP8, which are essential for the priming and activation of both the canonical NLRP3 and noncanonical CASP11-NLRP3 inflammasome (18). However, the roles of other canonical NLRP3 inflammasome regulatory factors in CASP11-NLRP3 inflammasome activation are largely unknown. One such regulator is caspase-6 (CASP6), an understudied caspase with a recently identified role in the activation of the ZBP1-NLRP3 inflammasome in response to influenza A virus (IAV) (19). However, the role of CASP6 in response to gram-negative bacterial infection has not been investigated. In this study, we found that during gram-negative bacterial infections, CASP6 plays a critical role in promoting CASP11-NLRP3 inflammasome activation. These results demonstrate that CASP6 promotes activation of the CASP11-NLRP3 inflammasome to control inflammatory cytokine production and cell death, key aspects of host defense, during gram-negative bacterial infections.

## Results

### CASP6 enhances CASP11-NLRP3 inflammasome activation during gram-negative bacterial infections

**Figure 1:**
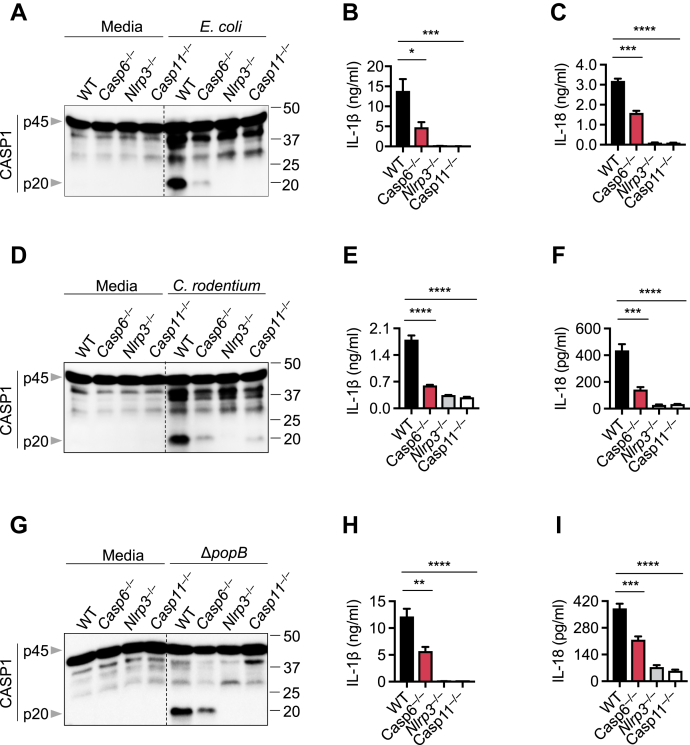
**CASP6 contributes to CASP11-NLRP3 inflammasome activation**. *A*, the immunoblot analysis of pro- (p45) and cleaved caspase-1 (p20; CASP1) in bone marrow-derived macrophages (BMDMs) after *Escherichia coli* infection at an MOI of 20 for 20 h. *B* and *C*, IL-1β (*B*) and IL-18 (*C*) release from BMDMs infected with *E. coli* at an MOI of 20 for 20 h. *D*, immunoblot analysis of pro- and cleaved CASP1 in BMDMs after *Citrobacter rodentium* infection at an MOI of 20 for 20 h. *E* and *F*, IL-1β (*E*) and IL-18 (*F*) release from BMDMs infected with *C. rodentium* at an MOI of 20 for 20 h. *G*, immunoblot analysis of pro- and cleaved CASP1 in BMDMs after *Pseudomonas aeruginosa* mutant strain *ΔpopB* infection at an MOI of 20 for 20 h. *H* and *I*, IL-1β (*H*) and IL-18 (*I*) release from BMDMs infected with *ΔpopB* at an MOI of 20 for 20 h. The data are representative of at least three independent experiments. The data are shown as mean ± SEM (*B*, *C*, *E*, *F*, *H*, and *I*). ∗*p* < 0.05, ∗∗*p* < 0.01, ∗∗∗*p* < 0.001, and ∗∗∗∗*p* < 0.0001 (one-way ANOVA). CASP, caspase; MOI, multiplicity of infection; NLRP, nucleotide-binding oligomerization domain-like receptor containing pyrin domain.

CASP6 has recently been shown to promote activation of the ZBP1-NLRP3 inflammasome in response to IAV infection (19). To investigate whether CASP6 also has a role in the regulation of the CASP11-NLRP3 inflammasome during gram-negative bacterial infections, we infected bone marrow-derived macrophages (BMDMs) isolated from WT and *Casp6*^−/−^ mice with the enteric bacteria *Escherichia coli* or *Citrobacter rodentium*. Loss of CASP6 resulted in reduced CASP1 activation following *E. coli* infection (Fig. 1*A*). In addition, the release of inflammasome-dependent cytokines IL-1β and IL-18 was significantly decreased in *Casp6*^−/−^ BMDMs compared with WT BMDMs (Fig. 1, *B* and *C*). These results suggest that CASP6 contributes to *E. coli*-induced CASP11-NLRP3 inflammasome activation. Similarly, we found that deleting CASP6 dampened the activation of the CASP11-NLRP3 inflammasome (Fig. 1*D*) and impaired the maturation of IL-1β and IL-18 (Fig. 1, *E* and *F*) in response to *C. rodentium* infection. In addition to these enteric bacteria, the *Pseudomonas aeruginosa* mutant strain Δ*popB* can also activate the CASP11-NLRP3 inflammasome (16, 20). To test whether CASP6 is involved in Δ*popB*-induced CASP11-NLRP3 inflammasome activation, we infected WT and *Casp6*^−/−^ BMDMs with *P. aeruginosa* Δ*popB*. Loss of CASP6 reduced CASP1 activation and IL-1β and IL-18 maturation in response to *P. aeruginosa* Δ*popB* (Fig. 1, *G*–*I*). Together, these results suggest that CASP6 is required for optimal CASP11-NLRP3 inflammasome activation following gram-negative bacterial infections.

### CASP6 does not regulate the expression of CASP11-NLRP3 inflammasome components during gram-negative bacterial infection

**Figure 2:**
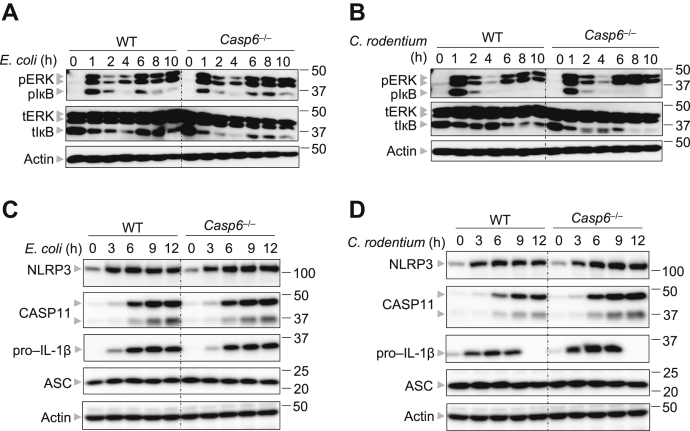
**CASP6 is not involved in priming the CASP11-NLRP3 inflammasome during gram-negative bacterial infections**. *A*, immunoblot analysis of phosphorylated ERK (pERK), phosphorylated IκB (pIκB), total ERK (tERK), and total IκB (tIκB) in bone marrow-derived macrophages (BMDMs) after *Escherichia coli* (20 MOI) infection for the indicated time. Actin was used as the internal control. *B*, immunoblot analysis of pERK, pIκB, tERK, and tIκB in BMDMs after *Citrobacter rodentium* (20 MOI) infection for the indicated time. Actin was used as the internal control. *C*, immunoblot analysis of NLRP3, CASP11, pro–IL-1β, and ASC in BMDMs after *E. coli* (20 MOI) infection for the indicated time. Actin was used as the internal control. *D*, immunoblot analysis of NLRP3, CASP11, pro–IL-1β, and ASC in BMDMs after *C. rodentium* (20 MOI) infection for the indicated time. Actin was used as the internal control. The data are representative of at least three independent experiments. ASC, apoptosis-associated speck-like protein containing a CARD; CASP, caspase; ERK, extracellular signal-regulated kinase; MOI, multiplicity of infection; NLRP, nucleotide-binding oligomerization domain-like receptor containing pyrin domain.

Assembly of the NLRP3 inflammasome traditionally requires two steps: priming and activation. Although the priming step is required for transcriptional upregulation of NLRP3 and pro–IL-1β, the activation step initiates NLRP3 oligomerization and subsequent inflammasome assembly (21). Given that the upregulation of NLRP3 and pro–IL-1β largely depends on the extracellular signal-regulated kinase (ERK) and NF-κB signaling pathways (1), we investigated the impact of CASP6 loss on the activation of these two signaling pathways following *E. coli* or *C. rodentium* infection. We observed comparable activation of ERK and NF-κB in WT and *Casp6*^−/−^ BMDMs upon infection with *E. coli* or *C. rodentium* (Fig. 2, *A* and *B*), suggesting that CASP6 is dispensable for the priming step of gram-negative bacterial infection-induced CASP11-NLRP3 inflammasome activation. To further confirm whether CASP6 had an effect on *Nlrp3* or *Il1b* expression, we conducted real-time qPCR to evaluate the mRNA expression levels of these two genes. In response to *E. coli* infection, neither *Nlrp3* nor *Il1b* mRNA levels were decreased in *Casp6*^−/−^ BMDMs compared with those in WT BMDMs (Fig. S1, *A* and *B*). It is also known that type I IFNs are essential for gram-negative bacteria-induced CASP11-NLRP3 inflammasome activation: type I IFNs are required for CASP11 upregulation (10), as well as the induction of IRGB10, *via* IRF1, to target the bacterial cell wall and release LPS for sensing (12). To evaluate whether CASP6 contributes to the expression of type I IFNs or IFN inducible genes, we assessed the mRNA expression of *Ifnb*, *Casp11*, *Irf1*, and *Irgb10* in *E. coli*-infected BMDMs. In line with the previous findings that apoptotic caspases suppress type I IFN production (22), we observed that the loss of CASP6 increased the expression of *Ifnb* in response to *E. coli* infection (Fig. S1*C*). Furthermore, the expression of IFN-inducible genes, *Casp11*, *Irf1*, and *Irgb10*, was not decreased in *E. coli*-infected *Casp6*^−/−^ BMDMs (Fig. S1, *D*–*F*). Similarly, we also observed the same trends for *Nlrp3*, *Il1b*, *Ifnb*, *Casp11*, *Irf1*, and *Irgb10* expression in *C. rodentium* infected BMDMs (Fig. S1, *G–L*). To further confirm that deleting CASP6 does not attenuate the expression of CASP11-NLRP3 inflammasome components, we analyzed protein expression. Consistent with the mRNA levels, the protein expression of NLRP3, CASP11, and pro–IL-1β was not impaired in *E. coli* or *C. rodentium-*infected *Casp6*^−/−^ BMDMs (Fig. 2, *C* and *D*). In addition, the expression of the adapter protein of the CASP11-NLRP3 inflammasome, ASC, was comparable between WT and *Casp6*^−/−^ BMDMs during both *E. coli* and *C. rodentium* infections (Fig. 2, *C* and *D*). Overall, our data suggest that CASP6 does not regulate the expression of the components of the CASP11-NLRP3 inflammasome during gram-negative bacterial infection.

### CASP6 regulates gram-negative bacteria-induced pyroptosis

CASP11 sensing of LPS is essential for CASP11 activation and the subsequent initiation of the CASP11-NLRP3 inflammasome assembly (4, 5, 6, 7). To determine whether CASP6 facilitates the interaction between CASP11 and LPS, we first tested the role of CASP6 in the activation of CASP11 during LPS transfection. There was no difference in CASP11 activation between WT and *Casp6*^−/−^ BMDMs (Fig. S2*A*). We next performed an LPS pulldown using a biotin-labeled LPS and streptavidin beads. We confirmed that CASP11 could be pulled down by LPS and that CASP6 did not show any binding affinity for LPS under these conditions (Fig. S2*B*). We then probed lysates from primary BMDMs and found that the binding of CASP11 to LPS was comparable between WT and *Casp6*^−/−^ BMDMs (Fig. S2*C*), suggesting that CASP6 has no role in the process of CASP11 binding to LPS.

**Figure 3:**
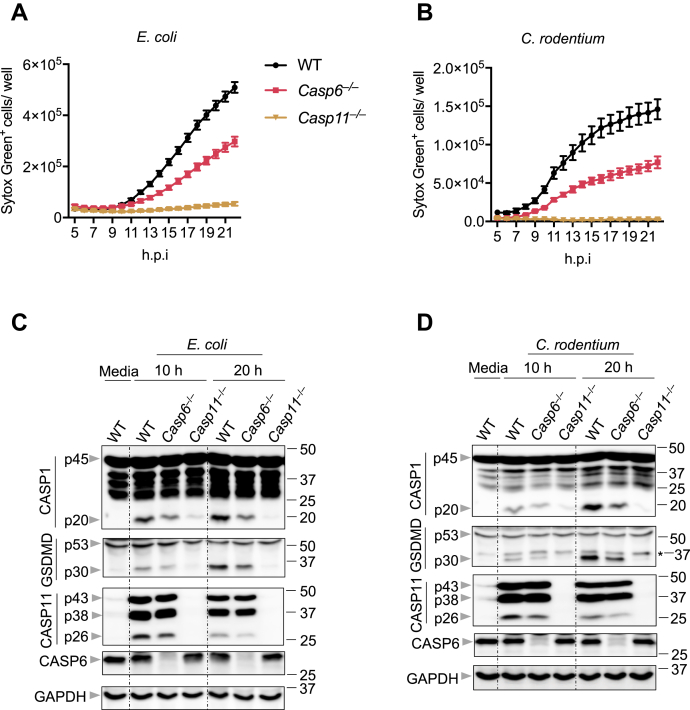
**CASP6 regulates pyroptosis during gram-negative bacterial infections**. *A* and *B*, real-time analysis of cell death in bone marrow-derived macrophages (BMDMs) using the IncuCyte imaging system and SYTOX Green nucleic acid staining after infection with *Escherichia coli* (20 MOI) (*A*) or *Citrobacter rodentium* (20 MOI) (*B*). quantification of the cell death at the indicated timepoints is shown. *C*, immunoblot analysis of pro- (p45) and cleaved caspase-1 (p20; CASP1), pro- (p53) and cleaved gasdermin D (p30; GSDMD), and pro- (p43) and cleaved caspase-11 (p38 and p26; CASP11) and caspase-6 (CASP6) in BMDMs after *E. coli* (20 MOI) infection for the indicated time. GAPDH was used as the internal control. *D*, immunoblot analysis of pro- and cleaved CASP1, pro- and cleaved GSDMD, and pro- and cleaved CASP11 and CASP6 in BMDMs after *C. rodentium* (20 MOI) infection for the indicated time. *Asterisk* indicates a nonspecific band. GAPDH was used as the internal control. The data are representative of at least three independent experiments. The data are shown as mean ± SEM (*A* and *B*). CASP, caspase; MOI, multiplicity of infection.

Combined with our data showing that CASP6 does not impact the priming of the CASP11-NLRP3 inflammasome or the expression of IRGB10, a key molecule involved in releasing LPS from the bacteria (12) (Figs. 2 and S1), these data suggest that the function of CASP6 may occur after CASP11 senses and binds LPS during gram-negative bacterial infection. Therefore, we investigated the role of CASP6 further downstream. Gram-negative bacterial infection induces cell death that is dependent on CASP11 leading to the cleavage of GSDMD to release its N-terminus and form pores in the plasma membrane (8, 9, 23, 24). The assembly of the CASP11-NLRP3 inflammasome occurs downstream of the GSDMD pore formation (8). To determine where CASP6 functions in this pathway, we next evaluated cell death in *Casp6*^−/−^ BMDMs after *E. coli* or *C. rodentium* infection. We observed that cell death was reduced in *Casp6*^−/−^ BMDMs compared with that in WT BMDMs in response to either *E. coli* or *C. rodentium* infection (Fig. 3, *A* and *B*), suggesting that GSDMD activation may be reduced in *Casp6*^−/−^ BMDMs. To confirm this, we evaluated the GSDMD cleavage in *E. coli-* or *C. rodentium-*infected BMDMs and found that the active form of GSDMD (p30) was reduced in *Casp6*^−/−^ BMDMs infected for 10 and 20 h (Fig. 3, *C* and *D*). In addition, CASP1 activation was attenuated in *Casp6*^−/−^ BMDMs infected with either *E. coli* or *C. rodentium* (Fig. 3, *C* and *D*). These data suggest that CASP6 acts upstream of the GSDMD pore formation.

To investigate the possibility that CASP6 could directly activate GSDMD, we overexpressed CASP6 together with GSDMD in 293T cells, using CASP11 as a positive control and uncleavable CASP6 (CASP6-DA) as a negative control. Overexpression of CASP11 with GSDMD resulted in the cleavage of GSDMD; however, the overexpression of CASP6 with GSDMD had no effect on GSDMD activation (Fig. S2*D*), indicating that CASP6 did not directly process GSDMD.

Because GSDMD maturation depends on the activity of CASP11 (24), we next evaluated whether CASP6 had any role in the processing of CASP11. We found that the formation of the p26 cleavage fragment of CASP11 was reduced in *Casp6*^−/−^ BMDMs infected with either *E. coli* or *C. rodentium* for 10 and 20 h (Fig. 3, *C* and *D*), suggesting that CASP6 is involved in the processing of CASP11 for its activation during gram-negative bacterial infection. Together, our results suggest that CASP6 contributes to CASP11 activation, thereby regulating the activation of pyroptosis during gram-negative bacterial infection.

### Catalytic activity of CASP6 contributes to CASP11-NLRP3 inflammasome activation during gram-negative bacterial infection

**Figure 4:**
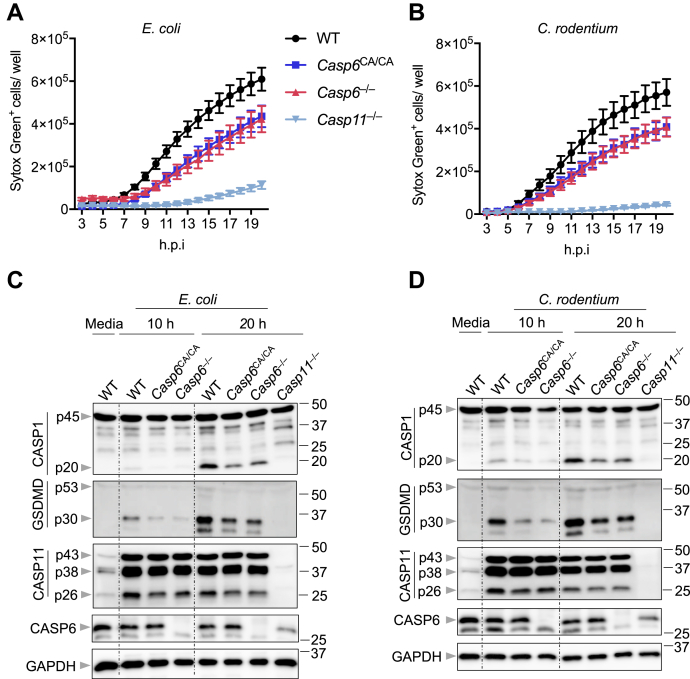
**Catalytic activity of CASP6 contributes to CASP11-NLRP3 inflammasome activation during gram-negative bacterial infections**. *A* and *B*, real-time analysis of cell death in bone marrow-derived macrophages (BMDMs) using the IncuCyte imaging system and SYTOX Green nucleic acid staining after infection with *Escherichia coli* (20 MOI) (*A*) or *Citrobacter rodentium* (20 MOI) (*B*). Quantification of the cell death at the indicated timepoints is shown. *C*, immunoblot analysis of pro- (p45) and cleaved caspase-1 (p20; CASP1), pro- (p53) and cleaved gasdermin D (p30; GSDMD), and pro- (p43) and cleaved caspase-11 (p38 and p26; CASP11) and caspase-6 (CASP6) in BMDMs after *E. coli* (20 MOI) infection for the indicated time. GAPDH was used as the internal control. *D*, immunoblot analysis of pro- and cleaved CASP1, pro- and cleaved GSDMD, and pro- and cleaved CASP11 and CASP6 in BMDMs after *C. rodentium* (20 MOI) infection for the indicated time. GAPDH was used as the internal control. The data are representative of at least three independent experiments. The data are shown as mean ± SEM (A and B). CASP, caspase; MOI, multiplicity of infection; NLRP, nucleotide-binding oligomerization domain-like receptor containing pyrin domain.

Because we observed that CASP6 contributed to the activation of CASP11 during gram-negative bacterial infection (Fig. 3, *C* and *D*), we hypothesized that the catalytic activity of CASP6 would be important in regulating the CASP11-NLRP3 inflammasome. To determine the effect of the catalytic activity of CASP6 during gram-negative bacterial infection, we established a mouse model carrying a mutation in CASP6 that created a catalytically dead version of the protein, *Casp6*^C146A/C146A^ (*Casp6*^CA/CA^). The identity of the mutation was confirmed by sequencing (Fig. S3). We then infected the *Casp6*^CA/CA^ BMDMs and monitored cell death. We found that both *E. coli* and *C. rodentium* infection-induced cell deaths were reduced in *Casp6*^CA/CA^ BMDMs, similar to the levels seen in *Casp6*^−/−^ BMDMs (Fig. 4, *A* and *B*). These results suggest that the catalytic activity of CASP6 is required for CASP6 regulation of gram-negative bacteria-induced cell death. In addition, CASP1 activation in *E. coli-* or *C. rodentium-*infected *Casp6*^CA/CA^ and *Casp6*^−/−^ BMDMs was comparable, and both were attenuated compared with the CASP1 activation in WT BMDMs (Fig. 4, *C* and *D*). Furthermore, GSDMD activation was reduced in both *Casp6*^CA/CA^ and *Casp6*^−/−^ BMDMs compared with that in WT BMDMs in response to *E. coli* and *C. rodentium* infections (Fig. 4, *C* and *D*). The activation of CASP11 was also decreased to similar levels in *Casp6*^CA/CA^ and *Casp6*^−/−^ BMDMs after *E. coli* or *C. rodentium* infections (Fig. 4, *C* and *D*). Collectively, our data here indicate that the catalytic activity of CASP6 contributes to gram-negative bacteria-induced cell death and CASP11-NLRP3 inflammasome activation.

### CASP6 cleaves CASP11

**Figure 5:**
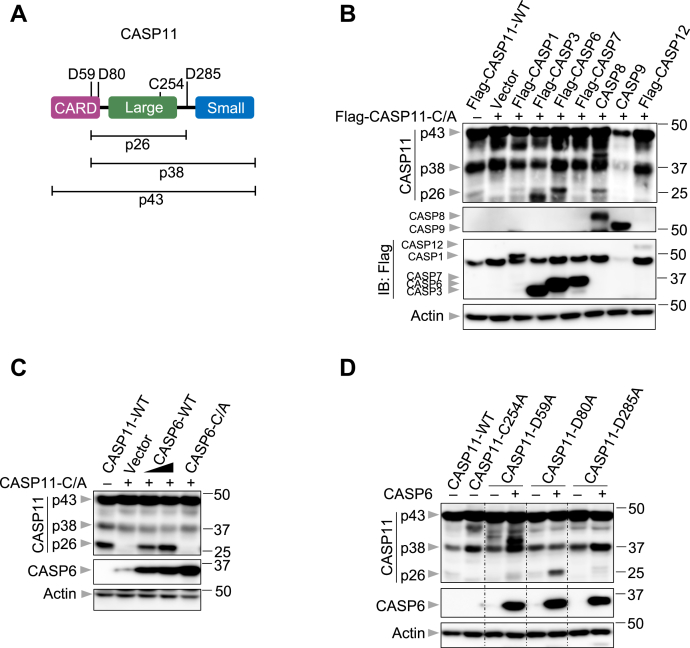
**CASP6 cleaves CASP11 in cell lysates**. *A*, schematic diagram of the cleavage and catalytic sites of caspase-11 (CASP11). p26, p38, and p43 indicate the fragments generated from the potential cleavage sites. *B*, immunoblot analysis of pro- (p43) and cleaved CASP11 (p38 and p26), caspase-8 (CASP8), caspase-9 (CASP9), caspase-12 (CASP12), caspase-1 (CASP1), caspase-7 (CASP7), caspase-6 (CASP6), and caspase-3 (CASP3) in 293T cells transfected with the indicated expression plasmids. Actin was used as the internal control. *C*, immunoblot analysis of pro- and cleaved CASP11 and CASP6 in 293T cells transfected with the indicated expression plasmids. Actin was used as the internal control. *D*, immunoblot analysis of pro- and cleaved CASP11 and CASP6 in 293T cells transfected with the indicated expression plasmids. Actin was used as the internal control. The data are representative of at least three independent experiments. CARD, caspase-activation and recruitment domain; CASP, caspase.

Because the catalytic activity of CASP6 contributed to CASP11 activation (Fig. 4, *C* and *D*), we hypothesized that CASP6 may be directly involved in CASP11 processing. CASP11 has several cleavage sites, and cleavage at different combinations of these sites yields products of differing sizes (Fig. 5*A*) (25). To investigate in an unbiased way whether CASP6 processes CASP11 directly, we used an overexpression system in which different caspase proteins were coexpressed with a catalytically dead version of CASP11 (CASP11-C/A). Overexpressing WT CASP11 alone resulted in its autoactivation to produce the p26 fragment, but the catalytically dead mutant did not produce the p26 fragment when overexpressed alone (Fig. 5*B*, Vector lane). We found that the p26 fragment was produced when CASP1, CASP3, CASP6, or CASP8 were individually coexpressed with the catalytically dead CASP11 (Fig. 5*B*). CASP3 expression also led to the cleavage of CASP11 at alternative cleavage sites, yielding a product smaller than p26, while coexpression of CASP6 and CASP11 increased the levels of the p26 fragment produced comparatively (Fig. 5*B*). By contrast, the overexpression of CASP7, CASP9, or CASP12 did not produce any fragments around 26 kD (Fig. 5*B*). To further confirm the role of CASP6 in CASP11 processing, we titrated the expression of CASP6. As CASP6 expression increased, the production of the CASP11 p26 fragment also increased (Fig. 5*C*), suggesting a dose-dependent effect of CASP6 on CASP11 processing. We also observed that the catalytic activity of CASP6 was required for the production of the CASP11 p26 fragment (Fig. 5*C*). To investigate the cleavage sites of CASP11 that were cleaved by CASP6, we constructed three different CASP11 mutants with the potential cleavage sites altered. Substitution of Asp80 with Ala resulted in robust processing of CASP11 and formation of the p26 fragment (Fig. 5*D*), as this cleavage site is not expected to be important to form the p26 fragment (Fig. 5*A*). However, mutating Asp59 to Ala in CASP11 reduced the comparative production of the p26 fragment and led to cleavage at alternative sites (Fig. 5*D*), suggesting that CASP6 can cleave after Asp59 in CASP11. Similarly, mutating Asp285 also reduced the amount of the p26 fragment produced (Fig. 5*D*). Together, these data suggest that CASP6 directly processes CASP11 *via* its cleavage sites, Asp59 and Asp285, in cell lysates.

## Discussion

CASP6 regulates NLRP3 inflammasome activation and PANoptosis during IAV infection by interacting with receptor-interacting protein kinase 3 (19). However, CASP6 is dispensable for absent in melanoma 2, NLR-family CARD-containing 4, and pyrin inflammasome activation, as well as for CASP11-NLRP3 inflammasome activation in response to chemical ligands and LPS transfection (19). In this study, we discovered that CASP6 contributes to gram-negative bacterial infection-induced CASP11-NLRP3 inflammasome activation. The loss of CASP6 led to decreased NLRP3 inflammasome activation, CASP11 and GSDMD cleavage, and cell death. The expression of the major components of the CASP11-NLRP3 inflammasome, including NLRP3, ASC, CASP1, and CASP11 was not affected in the absence of CASP6, suggesting that CASP6 is not involved in the priming step for CASP11-NLRP3 inflammasome assembly. Cells expressing the catalytically dead CASP6 mutant displayed similar phenotypes to those where CASP6 was deleted, indicating that CASP6 regulates the CASP11-NLRP3 inflammasome *via* its caspase activity.

Type I IFNs and IRFs are known to be key regulators in CASP11-NLRP3 inflammasome activation, as the expression of CASP11 is upregulated in response to IFNs, and IFNs drive IRGB10-mediated release of LPS from bacterial cell walls for sensing by CASP11 (10, 12, 16). The loss of apoptotic caspases has previously been shown to increase the expression of type I IFNs (22), and indeed, we detected increased type I IFN expression in *Casp6*^−/−^ BMDMs upon infection with gram-negative bacteria. However, we did not observe a change in the expression of *Irgb10*. CASP11-NLRP3 inflammasome activation can also be controlled through IRGB10- and GBP-independent mechanisms (16), and further studies will be needed to determine the specific roles of each of these proteins in the CASP6–CASP11–NLRP3 inflammasome axis.

We observed that the catalytic activity of CASP6 was required to regulate CASP11 activation, and our data suggest that CASP6 directly processes CASP11 to facilitate its activation. Whereas purified CASP11 can undergo autoprocessing for activation after binding to LPS (25), it is not clear whether other host factors are involved in CASP11 activation after it binds with LPS under physiological conditions in the context of infection with live bacteria. Our findings suggest that CASP6 has a critical role during infection, in contrast to it being dispensable during LPS transfection (19). It is well known that the basal levels of CASP11 are low with expression gradually increasing during infection, and it is possible that CASP11 needs additional factors to facilitate its activation when the expression levels are not sufficient. Additional studies are required to determine the threshold of CASP11 expression required for this autoactivation.

Our data also indicate that CASP8 could process CASP11 in the overexpression system, although processing by CASP8 is weaker than that by CASP6. Furthermore, CASP8 is a key upstream regulator of the activation of the CASP11-NLRP3 inflammasome (18), and CASP8 is activated during gram-negative bacterial infections (16). CASP8 can also directly cleave CASP6 to its active form (26). Therefore, it is possible that the CASP11 p26 fragment produced in CASP8-expressing cells was formed by endogenous CASP6 that was activated by CASP8 or by CASP8 itself. Additional studies are needed to investigate the detailed mechanism in this process.

Traditionally, CASP6 has been viewed as an apoptotic caspase. However, the increasing evidence has shown that CASP6 is also critical for other nonapoptotic processes (21, 27, 28, 29). Our findings here demonstrate that CASP6 can regulate CASP11 activation to mediate the activity of the CASP11-NLRP3 inflammasome, cytokine release, and cell death, further extending our understanding of the biological roles of this mysterious caspase in infection and disease.

## Experimental procedures

### Mice

*Casp6*^−/−^ (19) (Jackson Laboratory, 006236), *Nlrp3*^−/−^ (30), and *Casp11*^−/−^ (23) mice have been described previously. *Casp6*^C146A/C146A^ mutant mice were established using CRISPR-Cas9 technology and direct embryo injection. Briefly, chemically modified sgRNAs (Synthego) were tested before the embryo injection for activity in mouse N2A cells stably expressing Cas9 and assayed by targeted next generation sequencing (NGS), as previously described (31). The resulting NGS data were analyzed using CRIS.py (32). Editing construct sequences and relevant primers are listed in Table S1. For animal model generation, ten 3–4-week-old C57BL/6N female mice from Envigo were super ovulated with 5 units of gonadotrophin each from pregnant mare's serum (ProSpec) and 48 h later with 5 units of human chorionic gonadotrophin each (Sigma). After overnight mating with C57BL/6J males (The Jackson Laboratory), the females were euthanized, and the zygotes were harvested from the ampullae. The protective cumulus cells were removed using hyaluronidase, and the zygotes were washed and graded for fertilization by observing the presence of two pronuclei. A mixture of the sgRNA, Cas9, and ssODN consisting of 60 ng/μl of Cas9 protein (St Jude Protein Production Core), 30 ng/μl of sgRNA (Synthego), and 10 ng/μl of each ssODN (IDT) were infected into a pronucleus of zygotes. The injected zygotes were then returned to culture media (M16 from Millipore or A-KSOM from Millipore) and later, the same day transferred to 0.5 dpc pseudopregnant fosters (7–10 weeks old CD-1 females from Charles River Laboratories mated to vasectomized B6CBAF1 males from The Jackson Laboratory). Pups were born after 19 days gestation and sampled at day 10 to 14 for genotyping *via* targeted NGS. The animals positive for the C146A modification were weaned at day 21. After that, mice were backcrossed to the C57BL/6 genetic background mice at least three times. All mice were bred and housed at the Animal Resources Center in St Jude Children's Research Hospital and were backcrossed to the C57BL/6 background. All the experiments were carried out following protocols approved by the St Jude Children's Research Hospital Committee on the Use and Care of Animals.

### Bone marrow-derived macrophage culture and stimulation

Primary BMDMs from the indicated mouse were cultured for 6 days in IMDM (Thermo Fisher Scientific, 12440-053) supplemented with 10% fetal bovine serum (Biowest, S1620), 30% L929-conditioned medium, 1% nonessential amino acids (Thermo Fisher Scientific, 11140-050), and 1% penicillin and streptomycin (Thermo Fisher Scientific, 15070-063). BMDMs were seeded and incubated overnight with antibiotic-free BMDM media at a density of one million cells per well in 12-well plates before use. For bacterial infection, *E. coli*, *C. rodentium,* and *P. aeruginosa* Δ*popB* were used at an multiplicity of infection of 20. The cells were collected for protein or RNA analysis at the indicated timepoints. The supernatants were collected for ELISA.

### Immunoblot analysis

The cells were washed after infection and lysed with RIPA buffer plus protease and phosphatase inhibitors (Roche). For caspase-1 and gasdermin D immunoblotting, the cells were lysed together with the culture medium using lysis buffer containing protease inhibitors, phosphatase inhibitors, 10% NP-40, and 25 mM DTT. Proteins were then separated *via* 8%–12% SDS-PAGE and transferred to polyvinylidene fluoride membranes (Millipore, IPVH00010). After blocking with 5% skim milk, the membranes were incubated overnight at 4 °C with the following primary antibodies: anti-CASP1 (Adipogen, AG-20B-0042), anti-CASP6 (CST, #9762), anti-CASP8 (Enzo Life Science, #ALX-804-447-C100), anti-CASP9 (CST, #9504), anti-CASP11 (Novus Biologicals, NB120-10454), anti-NLRP3 (Adipogen, #AG-20B-0014), anti-ASC (Adipogen, #AG-25B-006-C100), anti-pro–IL-1β (CST, #12507), anti-GAPDH (CST, #5174), anti-GSDMD (Abcam, Ab209845), anti-pERK (CST, #9101), anti-pIκB (CST, #2859), anti-tERK (CST, #9102), anti-tIκB (CST, #9242), anti-HA (Millipore, 05-904), anti-Flag (Sigma, #F1804), and anti-β-actin (Proteintech, #66009-1-Ig). The membranes were washed three times with TBST and incubated with horse radish peroxidase-conjugated secondary antibodies (Jackson Immuno Research Laboratories) for 1 h. Protein bands were developed using Luminata Forte Western horse radish peroxidase substrate (Millipore, WBLUF0500), and the images were acquired using an Amersham Imager.

### Cytokine analysis

Cytokines were measured by multiplex ELISA (Millipore, MCYTOMAG-70K) or ELISA for IL-18 (BMS618-3; Invitrogen) according to the manufacturer's instructions.

### Real-time cell death analysis

Real-time cell death was analyzed using the two-color IncuCyte S3 incubator imaging system (Essence Biosciences). After bacterial infection, SYTOX Green (Life Technologies, S7020) was added into the cells and the images were obtained and analyzed using IncuCyte S3 software.

### Real-time quantitative PCR analysis

Following the bacterial infections, the cells were collected at the indicated timepoints for RNA extraction using TRIzol (Thermo Fisher Scientific, 15596026) according to the manufacturer's instructions. cDNA was synthesized with the isolated RNA using a First-Strand cDNA Synthesis Kit (Applied Biosystems, 4368814). Real-time PCR was conducted on an Applied Biosystems 7500 real-time PCR instrument using 2× SYBR Green (Applied Biosystems, 4368706). The primers used are as follows: *Gapdh*: 5′-CGT CCC GTA GAC AAA ATG GT-3′, 5′-TTG ATG GCA ACA ATC TCC AC-3′; *Il1b*: 5′-GAT CCA CAC TCT CCA GCT GCA-3′, 5′-CAA CCA ACA AGT GAT ATT CTC CAT G-3′; *Ifnb*: 5′-GCC TTT GCC ATC CAA GAG ATG C-3′, 5′-ACA CTG TCT GCT GGT GGA GTT C-3′; *Casp11*: 5′-ACA ATG CTG AAC GCA GTG AC-3′, 5′-CTG GTT CCT CCA TTT CCA GA-3′; *Nlrp3*: 5′-TCA GAT TGC TGT GTG TGG GAC TGA-3′, 5′- AGC TCA GAA CCA ATG CGA GAT CCT-3′; *Irf1*: 5′-GGC CGA TAC AAA GCA GGA GAA-3′, 5′-GGA GTT CAT GGC ACA ACG GA-3′; *Irgb10*: 5′-TAA TGC CCT TCG GGG AAT AGG-3′, 5′-CTG GTT TGA AGT TAG TTG TCC CA-3′. The expression is quantified relative to the expression of *Gapdh*.

### Transfection assays

293T cells were seeded into 24-well plates for overnight incubation. When the cells reached 80% confluence, the indicated plasmids (10 ng/well [low dose of CASP6-WT plasmid] or 100 ng/well [all others]) were transfected with Xfect polymer (Clontech Laboratories, 631318) according to the manufacturer's instructions. The samples were collected at 24 h post-transfection with RIPA buffer. For LPS transfection, the BMDMs were primed for 4 h with 500 ng/ml ultrapure LPS from *E. coli* (Invivogen, 0111:B4) and then transfected with 2 μg of LPS per well using Xfect polymer according to the manufacturer's instructions.

### Biotin-LPS pull-down assay

The pull-down assay was performed, as previously described (7). Briefly, 293T cells seeded in 6-well plates were transfected with the indicated plasmids using Xfect polymer according to the manufacturer's instructions. For primary BMDMs, the cells were primed with 100 ng/ml LPS for 4 h. At 48 h post-transfection or 4 h post-LPS stimulation, the cells were lysed with Triton buffer (50 mM Tris-HCl, pH 7.6, 150 mM NaCl, 2 mM EDTA, 1% Triton X-10, and protease inhibitor cocktail). After removing the cell debris, the supernatant was incubated with biotin-LPS (Invivogen, 0111:B4) and NeutrAvidin beads (Thermo Fisher Scientific, 29201) overnight at 4 °C. Then, the beads were washed three times with the Triton buffer, after which the precipitates were eluted with 2 ×SDS loading buffer and boiled for 5 min at 95 °C.

### Statistical analysis

GraphPad Prism 7.0 software was used for significance analysis. The data are presented as mean ± SEM. The one-way ANOVA with Dunnett's multiple comparisons test was used to determine the statistical significance. *p* values less than 0.05 were considered statistically significant where ∗*p* < 0.05, ∗∗*p* < 0.01, ∗∗∗*p* < 0.001, and ∗∗∗∗*p* < 0.0001.

## Data availability

All data generated for this study are included within this article.

## Supporting information

This article contains supporting information.

## Conflict of interest

The authors declare that they have no conflicts of interest with the contents of this article.
