# Supplementary material for: Caspase-6 promotes activation of the caspase-11-NLRP3 inflammasome during gram-negative bacterial infections
Source: J Biol Chem. 2021 Nov 2;297(6):101379. doi: 10.1016/j.jbc.2021.101379 (PMC8633687; doi:10.1016/j.jbc.2021.101379)
Supplement: Figures S1–S3 and Table S1 [file mmc1.pdf]

Supporting Information

Caspase-6 promotes activation of the caspase-11-NLRP3 inflammasome during gram-negative bacterial infections

Min Zheng[1], Rajendra Karki[1], Balabhaskararao Kancharana[1], Hartmut Berns[2], Shondra M. Pruett-Miller[2], and Thirumala-Devi Kanneganti[1]*

[1] Department of Immunology, [2] Center for Advanced Genome Engineering, St. Jude Children's Research Hospital, Memphis, TN, 38105, USA

SUPPLEMENTARY FIGURES AND TABLES

Figure S1. CASP6 deficiency does not affect the priming of the CASP11-NLRP3 inflammasome.

Figure S2. CASP6 is not involved in LPS binding and is not able to directly cleave GSDMD.

Figure S3. The catalytically dead CASP6 mutant mouse carries the homozygous C146A mutation.

Table S1. List of CRISPR/Cas9 gene editing construct sequences and relevant primers.

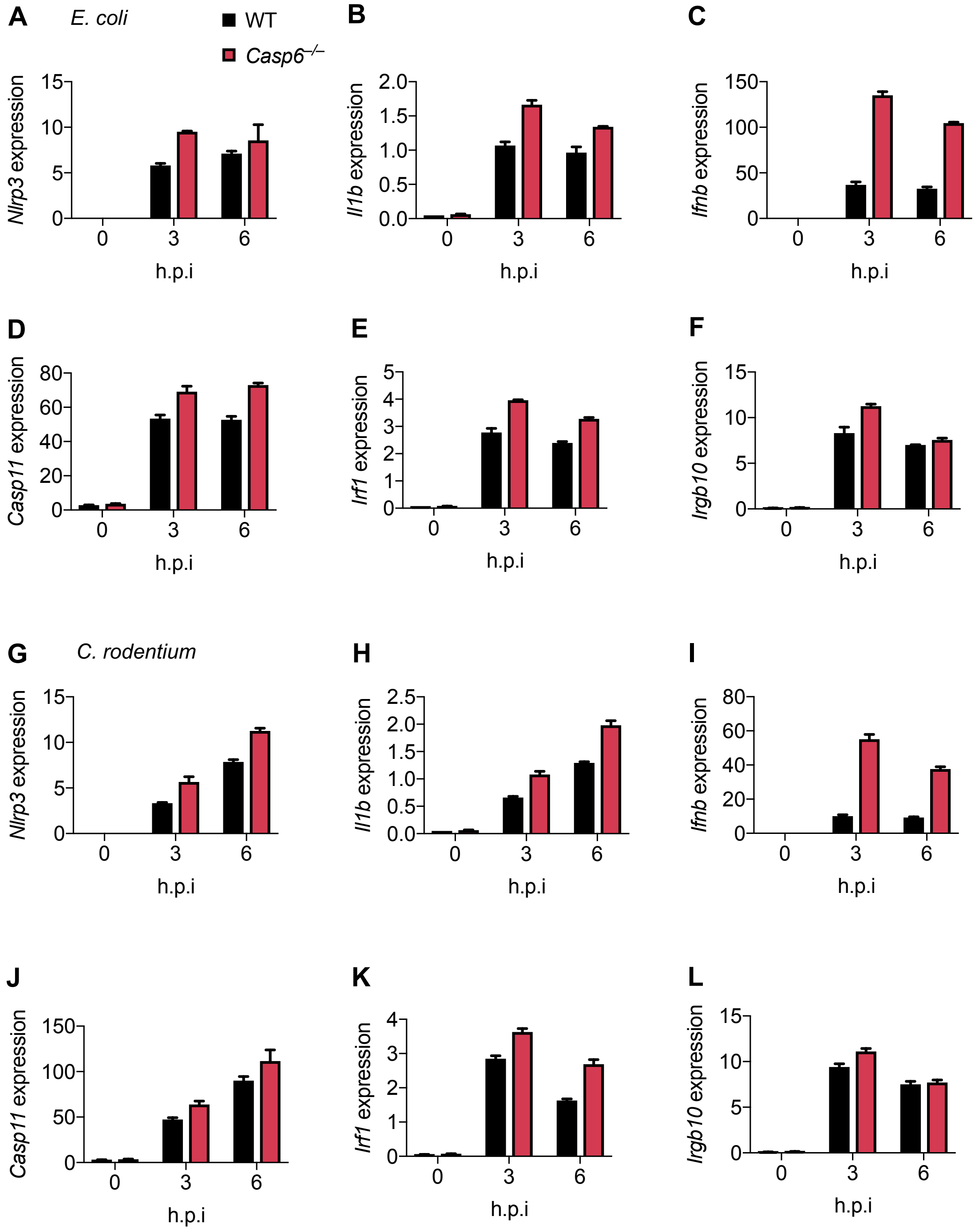

Figure S1. CASP6 deficiency does not affect the priming of the CASP11-NLRP3 inflammasome.

(A–F) Real time PCR analysis of the expression of *Nlrp3* (A), *Il1b* (B), *Ifnb* (C), *Casp11* (D), *Irf1* (E), and *Irgb10* (F) in bone marrow-derived macrophages (BMDMs) after infection with *E. coli* (20 MOI) for the indicated time. (G–L) Real time PCR analysis of the expression of *Nlrp3* (G), *Il1b* (H), *Ifnb* (I), *Casp11* (J), *Irf1* (K), and *Irgb10* (L) in BMDMs after infection with *C. rodentium* (20 MOI) for the indicated time. Data are representative of at least three independent experiments. Data are shown as mean ± SEM (A–L).

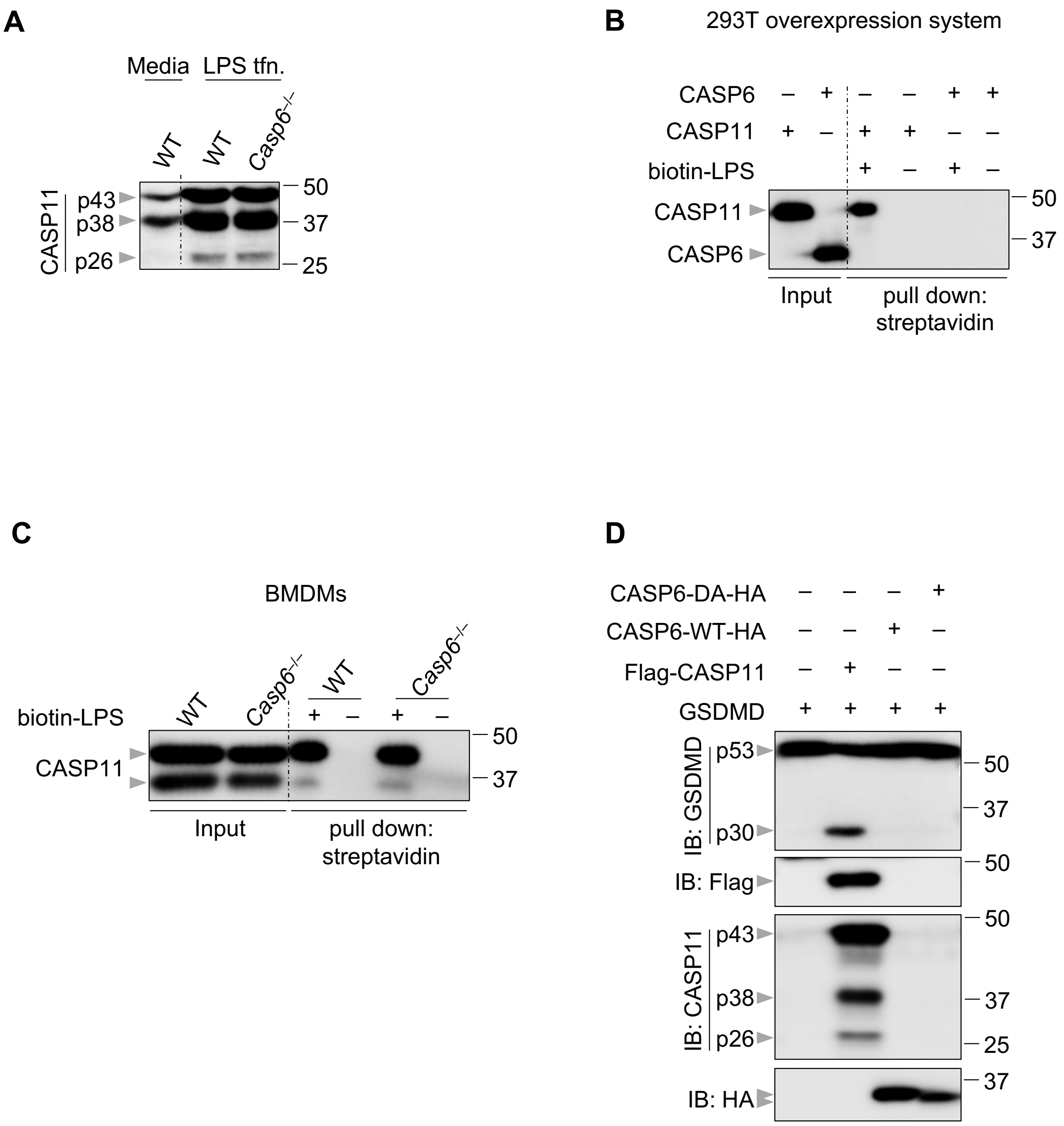

Figure S2. CASP6 is not involved in LPS binding and is not able to directly cleave GSDMD. (A) Pro- (p43) and cleaved (p38 and p26) caspase-11 (CASP11) in bone marrow-derived macrophages (BMDMs) transfected with LPS (LPS tfn.) for 4 h. (B) Biotin-streptavidin–mediated LPS pull down and total lysates from 293T cells transfected with the indicated expression plasmids. (C) Biotin-streptavidin–mediated LPS pull down and total lysates from LPS-primed BMDMs. (D) Immunoblot analysis of pro- (p53) and cleaved gasdermin D (p30; GSDMD), Flag-tagged CASP11, pro- (p43) and cleaved CASP11 (p38 and p26) and HA-tagged caspase-6 (CASP6). Data are representative of at least three independent experiments.

A

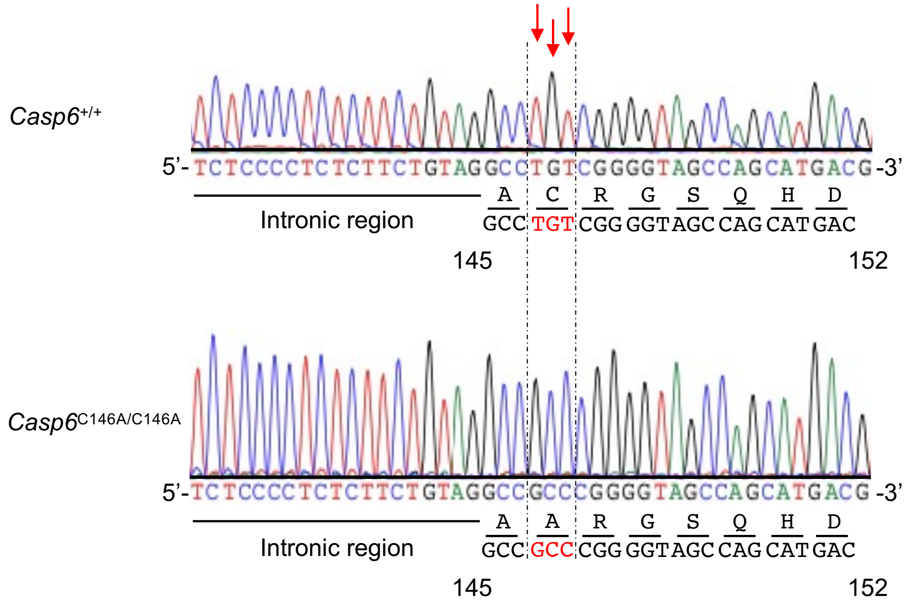

Figure S3. The catalytically dead CASP6 mutant mouse carries the homozygous C146A mutation.

(A) Sequencing results of samples from WT and *Casp6*$^{C146A/C146A}$ (*Casp6*$^{CA/CA}$) mice. The red arrows indicate the mutated nucleotides.

Table S1. List of CRISPR/Cas9 gene editing construct sequences and relevant primers.

Name	Sequence (5' to 3')
CAGE302.Casp6.g5 spacer	CCUCUCUUCUGUAGGCCUGU
CAGE289.g3.sense.ssODN *AltR modifications	*tgtccaggggaaccacgggtacgtcatgctggctaccccgGGCggcct acagaagagaggggagagggcacgttctctgcctg
CAGE302.DS.F	GCATCTCTCAGAGCCTGATTGCCTCG
CAGE302.DS.R	AGTAGCACATGAGGAAGTCTGCCCC
